# Impact of the preoperative prognostic nutritional index on survival outcomes in upper tract urothelial carcinomas

**DOI:** 10.1002/cam4.2161

**Published:** 2019-05-09

**Authors:** Wenbin Xue, Ping Tan, Hang Xu, Lu Yang, Qiang Wei

**Affiliations:** ^1^ Department of Urology & Institute of Urology West China Hospital, Sichuan University Chengdu China

**Keywords:** albumin, lymphocyte, prognostic nutritional index, radical nephroureterectomy, upper tract urothelial carcinoma

## Abstract

**Purposes:**

To investigate the value of prognostic nutritional index (PNI) in patients with upper tract urothelial carcinoma (UTUC) who underwent radical nephroureterectomy (RNU).

**Patients and methods:**

A total of 717 patients were included in our study from 2003 to 2016. PNI was calculated as 10 × serum albumin level (g/dL) + 0.005 × total lymphocyte count (per mm^3^). Kaplan‐Meier analysis and Cox regression models were adapted to analyze the value of PNI on survival outcomes.

**Results:**

The cutoff value of PNI was set as 46.91 and 298 patients (47.6%) had PNI <46.91. The median follow‐up was 50 months. The results suggested that low PNI was significantly associated with worse pathologic features (all *P* < 0.001). Multivariable Cox regression analysis revealed that PNI < 46.91 was an independent predictor of poor overall survival (Hazard ratios [HR] = 1.777, 95% CI = 1.383‐2.284, *P* < 0.001), cancer‐specific survival (HR = 1.850, 95% CI = 1.399‐2.445, *P* < 0.001), and recurrence‐free survival (HR = 1.554, 95% CI = 1.229‐1.964, *P* < 0.001).

**Conclusions:**

Low preoperative PNI was associated with worse survival outcomes in patients with UTUC. PNI could be an easily assessed blood‐based biomarker to predict the prognosis in patients with UTUC treated with RNU.

## 1 INTRODUCTION

Upper tract urothelial carcinoma (UTUC) is a relatively rare but potentially fatal disease, which occurs in the pyelocaliceal cavities or ureter. It accounts for 5%‐10% of urothelial carcinomas.^1^ Although the radical nephroureterectomy (RNU) with bladder cuff excision has been considered as the standard treatment for the patients with UTUCs, the prognosis remains poor with a potential of intravesical recurrence and distant metastasis.^2^ Despite the use of adjuvant chemotherapy, the overall survival (OS) of the patients has not been improved because of the complications.^3^, ^4^ Therefore, the identification of the prognostic factors is needed to improve therapies.

Till now, many preoperative and postoperative prognostic factors of UTUC have been indicated,^5^, ^6^ such as lymphovascular invasion (LVI), tumor stage, tumor grade, tumor size, and lymph node invasion,^7^, ^8^ which can be used to predict prognosis and adapt the treatment for the patients of UTUC. However, there are limited data about preoperative prognostic factor in UTUC. Recently, amounting evidence has suggested that patients' nutritional and immunologic conditions could influence the postoperative outcomes of malignant tumors, like breast cancer,^9^ nonsmall cell lung cancer,^10^ and colorectal cancer.^11^

The prognostic nutritional index (PNI), which was calculated based on serum albumin levels and total lymphocyte count, was first reported by Buzby and colleagues in 1980.^12^ To date, many studies have proved that PNI is a significant indicator for prognosis in patients with several malignancies, but the prognostic value of PNI has been poorly investigated in UTUC. Therefore, our study was designed to identify the impact of PNI on the survival and pathologic outcomes of patients with UTUC after RNU.

## 2 PATIENTS AND METHODS

### 2.1 Patient selection

A total of 806 patients with UTUC who underwent RNU from our institution were retrieved between January 2003 and December 2016. Patients with missing PNI data (n = 23), history of receiving preoperative chemotherapy or radiotherapy (n = 21), presence of inflammatory condition (n = 17), as well as those who were withdrawn within 3 months (n = 28) were excluded. Finally, 717 patients were included in the analyses. RNU was performed as standard procedure including the dissection of kidney with the entire part of ureter, and the bladder cuff resection. Lymphadenectomy was performed in the patients with enlarged lymph nodes which were indicated by preoperative radiology or intraoperative inspection.

### 2.2 Clinical and pathologic evaluation

Clinical features including patients' age, gender, surgical approach, smoking history, hydronephrosis, tumor size, and tumor side. Tumor stage was evaluated by the TNM classification system^13^ and tumor grade was assessed on the basis of the 1998 WHO consensus classification.^14^ LVI, multifocality, tumor architecture, and surgical margin status were reported by experienced urologic pathologists. The PNI data were extracted through the laboratory examination reports before surgery, which was calculated as 10 × serum albumin level (g/dL) + 0.005 × total lymphocyte count (per mm^3^).^15^

### 2.3 Follow‐up

Patients were assessed every 3 months for the first year and every 6 months for the second and third year after RNU. Then annually thereafter. Routine check‐ups included blood laboratory tests (blood routine examination, liver, and renal functions examination), medical history, cystoscopy, and imaging (chest/abdomen CT/MRI, carried out every year or if clinically indicated). Duration of follow‐up ranged from the date of operation to the latest follow‐up or death, which was defined as cancer related to the tumor or not.

### 2.4 Statistical analysis

All the patients were divided into two groups: patients with PNI ≥46.91 and patients with PNI <46.91. The cutoff value of PNI was defined as 46.91 according to the receiver operating characteristic (ROC) curves as well as Youden Index.^16^, ^17^ Student’s *t* test and chi‐squared test were adapted to analyze the continuous and categorical variables, respectively. Kaplan‐Meier curves were used to calculate cancer‐specific survival (CSS), recurrence‐free survival (RFS), and OS. The differences were assessed by using the log‐rank test. Univariable and multivariable Cox regression models were conducted to evaluate the risk factors for CSS, RFS, and OS, and those with *P* < 0.1 in the univariable model were accepted into the multivariable analyses. The multivariable Cox regression analysis was adjusted for tumor stage, tumor grade, tumor size, tumor architecture, surgical margin status, concomitant variant histology (CVH), lymph node status, LVI status, and PNI. Hazard ratios (HRs) were used to evaluate the strength of the variables with 95% CIs. The result of *P* < 0.05 was defined as statistical significance. All the analyses were conducted using SPSS 22.0 (IBM SPSS, Chicago, IL).

## 3 RESULTS

### 3.1 Characteristics of included patients

**Figure 1:**
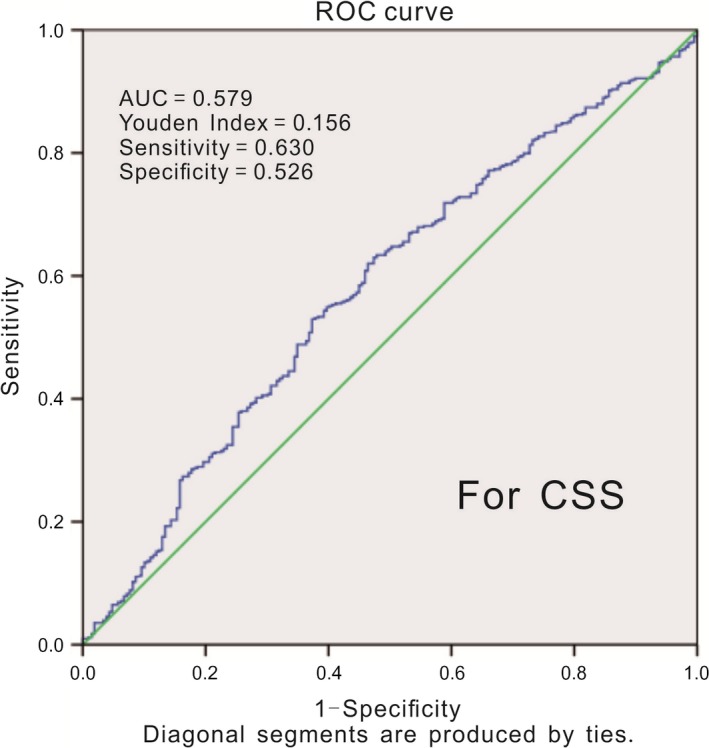
ROC curve of PNI of RFS in patients with UTUC and the cutoff of PNI was 46.91, with a sensitivity of 63% and a specificity of 52.6%

The characteristics of patients with UTUC in our study are presented in Table 1. Of all the 717 patients included, 298 were in PNI <46.91 group and 419 were in PNI ≥46.91 group. The cutoff of 46.91 was calculated by using the ROC curves (Figure 1). The median follow‐up duration was 50 months (interquartile range 28‐78 months). For the included patients, 408 (56.9%) were men and 309 (43.1%) were women. Four hundred and eighty‐four (67.5%) patients underwent open RNU and the remaining 233 (32.5%) patients underwent laparoscopic RNU. Among the patients, 205 (28.6%) had the tumor in the ureter, 385 (53.7%) had the tumor in the renal pelvis, and 127 (17.7%) had multifocal lesions. Pathological T stage was pTis/Ta/T1 in 221 cases (30.8%), pT2 in 145 (20.2%), pT3 in 248 (34.6%), and pT4 in 103 (14.4%). 71 (9.9%) patients were diagnosed with positive lymph nodes.

**Table 1:** Demographics and clinicopathological characteristics of patients with urinary tract urothelial carcinoma included in present study

Characteristic	Total	PNI ＜46.91 (n = 298, 47.6%)	PNI ≥ 46.91 (n = 419, 52.4%)	*P*
Gender (male vs female)	408/309	172/126	236/183	0.710
Age (>67 vs <67 years)	354/363	160/138	194/225	0.051
Body mass index (≥25 vs <25 kg/m^2^)	188/529	70/228	118/301	0.161
Smoking history (yes vs no)	204/513	89/209	115/304	0.479
Tumor side (right vs left)	350/367	154/144	196/223	0.196
Surgical approach, *n* (%)				0.646
Open RNU	484 (67.5)	204 (68.5)	280 (66.8)	
Laparoscopic RNU	233 (32.5)	94 (31.5)	139 (33.2)	
Hydronephrosis (Yes vs No)	447/270	177/121	270/149	0.170
Tumor location, *n* (%)				0.013
Pelvicalyceal	385 (53.7)	169 (56.7)	216 (51.6)	
Ureteric	205 (28.6)	69 (23.2)	136 (32.5)	
Both	127 (17.7)	60 (20.1)	67 (16.0)	
Tumor grade (High vs Low)	528/189	239/59	289/130	0.001
Tumor stage, *n* (%)				<0.001
Tis, Ta, T1	221 (30.8)	74 (24.8)	147 (35.1)	
T2	145 (20.2)	56 (18.8)	89 (21.2)	
T3	248 (34.6)	107 (35.9)	141 (33.7)	
T4	103 (14.4)	61 (20.5)	42 (10.0)	
Lymph node status, *n* (%)				0.434
pN0	90 (12.6)	41 (13.8)	49 (11.7)	
pNx	556 (77.5)	224 (75.2)	332 (79.2)	
pN+	71 (9.9)	33 (11.1)	38 (9.1)	
LVI (positive vs negative)	107/610	54/244	53/366	0.043
Tumor size (＞3 vs ≤3 cm)	488/229	207/91	281/138	0.497
Surgical margin status (positive vs negative)	58/659	26/272	32/387	0.599
Multifocality (present vs absent)	119/598	45/253	74/345	0.364
Sessile vs papillary	492/225	223/75	269/150	0.002
CVH (with vs without)	165/552	80/218	85/334	0.040
Bladder cancer status, *n* (%)				0.930
No	616 (85.9)	255 (85.6)	361 (86.2)	
Previous	22 (3.1)	10 (3.4)	12 (2.9)	
Concomitant	79 (11.0)	33 (11.1)	46 (11.0)	
Adjuvant therapy (yes vs no)	291/426	117/181	174/245	0.543
Serum albumin (g/L)	39.74 ± 5.03	35.53 ± 4.36	42.74 ± 2.85	<0.001
Lymphocyte count (10^9^)	1.73 ± 6.51	1.12 ± 0.43	2.17 ± 8.49	0.184

Abbreviations: RNU, radical nephroureterectomy; LVI, lymphovascular invasion; CVH, concomitant variant histology.

### 3.2 Low PNI (<46.91) independently predicted poor OS, RFS, and CSS

#### 3.2.1 Low PNI and OS

**Figure 2:**
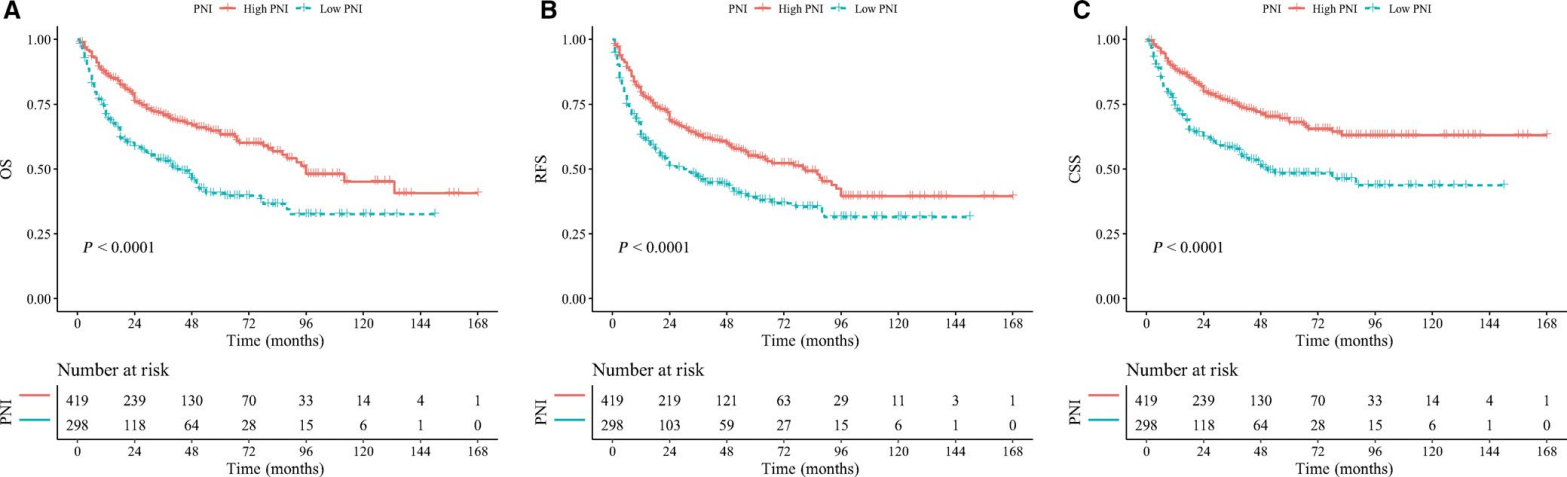
Kaplan‐Meier curves for OS (A), RFS (B), and CSS (C) which were performed according to PNI value for UTUC patients after RNU

During the follow‐up, 260 patients (36.3%) died of all causes, and the 3‐year and 5‐year OS were 70.8% and 63.3% for the high PNI group, as well as 53.8% and 40.6% for the low PNI group, respectively. Kaplan‐Meier survival analysis suggested that patients with low PNI had worse OS compared to those with high PNI (log‐rank test, *P* < 0.001) (Figure 2). Subsequently, our univariable analysis showed that patients with low PNI were statistically significantly correlated with worse OS (HR = 1.90, *P* < 0.001; Table 2). Meanwhile, multivariable analysis revealed that low PNI was a significant indicator of worse OS (HR = 1.78, *P* < 0.001; Table 3).

**Table 2:** Univariable Cox regression analyses of survival outcomes in patients with UTUC

Characteristic	Overall survival			Cancer‐specific survival			Recurrence‐free survival		
	HR	95% CI	*P*	HR	95% CI	*P*	HR	95% CI	*P*
Gender (male vs female)	1.141	0.894‐1.457	0.290	1.214	0.925‐1.593	0.163	1.167	0.929‐1.466	0.184
Age (>67 vs ≤67 years)	1.020	0.800‐1.302	0.872	0.928	0.707‐1.217	0.588	0.938	0.747‐1.177	0.581
BMI (≥25 vs < 25 kg/m^2^)	0.953	0.726‐1.251	0.728	0.878	0.645‐1.195	0.409	0.978	0.760‐1.259	0.864
Smoking history (yes vs no)	0.901	0.683‐1.188	0.458	0.862	0.631‐1.177	0.350	0.884	0.682‐1.144	0.348
Tumor side (right vs left)	1.051	0.824‐1.341	0.687	1.089	0.830‐1.428	0.538	1.063	0.847‐1.333	0.601
Surgical approach (open vs laparoscopic)	0.724	0.541‐0.969	0.030	0.677	0.490‐0.934	0.018	0.869	0.672‐1.124	0.285
Hydronephrosis (yes vs no)	1.342	1.035‐1.740	0.026	1.249	0.938‐1.664	0.128	1.401	1.097‐1.788	0.007
Tumor location, n (%)			0.712			0.537			0.547
Pelvicalyceal	1	Reference		1	Reference		1	Reference	
Ureteric	0.958	0.719‐1.277	0.771	1.012	0.737‐1.390	0.941	0.955	0.731‐1.249	0.738
Both	1.119	0.800‐1.565	0.510	1.224	0.849‐1.763	0.279	1.154	0.846‐1.574	0.366
Tumor grade (High vs Low)	2.832	1.991‐4.028	<0.001	3.471	2.268‐5.313	<0.001	2.276	1.679‐3.085	<0.001
Tumor stage, n (%)			<0.001			<0.001			<0.001
Tis, Ta, T1	1	Reference		1	Reference		1	Reference	
T2 vs Tis, Ta, T1	1.621	1.040‐2.529	0.033	1.598	0.951‐2.686	0.077	1.499	1.011‐2.223	0.044
T3 vs Tis, Ta, T1	3.279	2.266‐4.744	<0.001	3.602	2.355‐5.511	<0.001	2.836	2.041‐3.941	<0.001
T4 vs Tis, Ta, T1	7.984	5.374‐11.861	<0.001	9.293	5.938‐14.544	<0.001	6.974	4.857‐10.013	<0.001
Lymph node status, n (%)			<0.001			<0.001			<0.001
pN0	1	Reference		1	Reference		1	Reference	
pNx vs pN0	1.524	0.995‐2.336	0.053	1.516	0.928‐2.478	0.097	1.517	1.022‐2.252	0.039
pN+ vs pN0	5.392	3.295‐8.823	<0.001	6.068	3.504‐10.509	<0.001	5.496	3.467‐8.714	<0.001
LVI (positive vs negative)	2.452	1.841‐3.265	<0.001	2.666	1.950‐3.645	<0.001	2.177	1.652‐2.869	<0.001
Tumor size (>3 vs ≤3 cm)	1.988	1.494‐2.644	<0.001	2.029	1.473‐2.796	<0.001	1.862	1.433‐2.420	<0.001
Surgical margin status (positive vs negative)	2.147	1.482‐3.111	<0.001	2.370	1.591‐3.532	<0.001	1.898	1.321‐2.728	0.001
Multifocality (present vs absent)	0.922	0.658‐1.291	0.635	1.012	0.704‐1.456	0.947	0.947	0.694‐1.294	0.734
Sessile vs papillary	2.968	2.151‐4.095	<0.001	3.653	2.480‐5.380	<0.001	2.536	1.906‐3.374	<0.001
CVH (with vs without)	2.199	1.697‐2.850	<0.001	2.357	1.770‐3.138	<0.001	2.019	1.578‐2.584	<0.001
Bladder cancer status, n (%)			0.136			0.203			0.376
No	1	Reference		1	Reference		1	Reference	
Previous	0.297	0.074‐1.198	0.088	0.345	0.085‐1.391	0.134	0.903	0.425‐1.920	0.792
Concomitant	1.198	0.835‐1.719	0.327	1.205	0.809‐1.795	0.360	1.263	0.901‐1.770	0.176
Adjuvant therapy (Yes vs No)	0.858	0.671‐1.097	0.222	0.920	0.701‐1.209	0.551	1.090	0.868‐1.369	0.456
PNI (<46.91 vs ≥46.91)	1.895	1.485‐2.418	<0.001	2.014	1.534‐2.643	<0.001	1.635	1.302‐2.054	<0.001

Abbreviations: RNU, radical nephroureterectomy; LVI, lymphovascular invasion; CVH, concomitant variant histology.

**Table 3:** Multivariable Cox regression analysis of survival outcomes in patients with UTUC

Characteristic	Overall survival			Cancer‐specific survival			Recurrence‐free survival		
	HR	95% CI	*P*	HR	95% CI	*P*	HR	95% CI	*P*
Tumor grade (high vs low)	1.736	1.188‐2.537	0.004	1.944	1.235‐3.061	0.004	1.483	1.066‐2.064	0.019
Tumor stage, n (%)			<0.001			0.001			<0.001
Tis, Ta, T1	1	Reference		1	Reference		1	Reference	
T2 vs Tis, Ta, T1	1.203	0.757‐1.914	0.434	1.101	0.641‐1.893	0.727	1.168	0.774‐1.763	0.459
T3 vs Tis, Ta, T1	1.889	1.233‐2.895	0.003	1.860	1.142‐3.028	0.013	1.834	1.256‐2.679	0.002
T4 vs Tis, Ta, T1	2.837	1.710‐4.705	<0.001	2.723	1.536‐4.828	0.001	3.008	1.892‐4.780	<0.001
Lymph node status, n (%)			<0.001			<0.001			<0.001
pN0	1	Reference		1	Reference		1	Reference	
pNx vs pN0	2.005	1.297‐3.098	0.002	1.995	1.212‐3.284	0.007	1.930	1.294‐2.880	0.001
pN+ vs pN0	3.174	1.877‐5.367	<0.001	3.460	1.933‐6.193	<0.001	3.348	2.043‐5.486	<0.001
LVI (positive vs negative)	1.079	0.783‐1.487	0.643	1.101	0.778‐1.560	0.586	0.961	0.702‐1.315	0.801
Tumor size (>3 vs ≤3cm)	1.717	1.273‐2.318	<0.001	1.717	1.226‐2.404	0.002	1.603	1.220‐2.106	0.001
Surgical margin status (positive vs negative)	1.126	0.762‐1.662	0.552	1.191	0.785‐1.809	0.411	1.046	0.713‐1.533	0.819
Sessile vs papillary	1.520	1.043‐2.215	0.029	1.737	1.110‐2.718	0.016	1.415	1.011‐1.979	0.043
CVH (with vs without)	1.392	1.060‐1.827	0.017	1.435	1.064‐1.934	0.018	1.291	0.996‐1.674	0.054
PNI (<46.91 vs ≥46.91)	1.777	1.383‐2.284	<0.001	1.850	1.399‐2.445	<0.001	1.554	1.229‐1.964	<0.001

Abbreviations: RNU, radical nephroureterectomy; LVI, lymphovascular invasion; CVH, concomitant variant histology.

#### 3.2.2 Low PNI and RFS

The 3‐year and 5‐year RFS were 63.0% and 55.2% for the high PNI group, and 46.6% and 39.1% for the low PNI group, respectively. The Kaplan‐Meier curve proved that the rate of disease recurrence was higher in the low PNI group than that in the high PNI group (*P* < 0.001) (Figure 2). Moreover, univariable Cox regression analysis suggested that low PNI was significantly associated with the higher rate of disease recurrence (HR = 1.64, *P* < 0.001; Table 2). Low PNI was also indicated as a significant indicator of poor RFS through the multivariable Cox regression analysis (HR = 1.55, *P* < 0.001; Table 3).

#### 3.2.3 Low PNI and CSS

A total of 209 patients (29.1%) died from cancer during follow‐up, and 3‐year and 5‐year CSS were 58.4% and 48.3% for the low PNI group and 75.5% and 68.1% for the high PNI group, respectively. Patients with low PNI had a significant worse CSS rate (*P* < 0.001) compared to the patients with high PNI according to the Kaplan‐Meier survival curve (Figure 2). Univariable analysis revealed that low PNI was significantly correlated with unfavorable CSS (HR = 2.01, *P* < 0.001). At the same time, multivariable analysis showed low PNI was a significant prognostic factor for poorer CSS (HR = 1.85, *P* < 0.001; Table 2).

Furthermore, our analysis also suggested that high tumor grade, tumor stage of T3 or T4, lymph node invasion, CVH, tumor size ≥3 cm, and sessile carcinoma also correlated with poor OS, RFS, and CSS (all *P* < 0.05; Table 3).

## 4 DISCUSSION

In our study, we found that PNI was a significant predictor for worse pathologic and oncologic outcomes in patients with UTUC. Comparing with the patients with high PNI, those with low PNI had decreased OS, RFS, and CSS. In the multivariable analysis, we found that PNI was an independent prognostic factor for OS, RFS, and CSS in UTUC.

PNI was first performed as a predictive indicator by Buzby and colleagues,^12^ who reported a complex formula as: PNI = 158‐0.78 × triceps skinfold (mm) – 16.6 × albumin (g/100 mL) – 5.8 × cutaneous delayed hypersensitivity – 0.20 × transferrin (mg/100 mL). In contrast, Onodera and coworkers^15^ calculated the PNI based on the total lymphocyte count and the serum albumin levels, which were more easily assessable. In our study, we used the latter method, and the ROC curve analysis suggested the cutoff value of PNI was 46.91. When the PNI was 46.91, the specificity and sensitivity for the 5‐year CSS were 52.6% and 63.0%, respectively.

PNI, a combination of serum albumin and lymphocyte count, has been reported as a useful predictor in several malignancies (eg. Lung cancer,^10^ breast cancer,^9^ colorectal cancer,^11^ and renal cell carcinoma 18). To date, we found that only a single study, which was conducted by Huang et al in 2017,^19^ had reported the prognostic value of PNI in UTUC. Four hundred and twenty‐five patients were included in their study and the results showed that PNI was a useful independent predictor for patients with UTUCs, which was consistent with our findings. In our analysis, we had a larger sample size and included more indicators, which was helpful for risk prediction in UTUCs.

Recently, a growing body of literature revealed that cancer‐related malnutrition had a negative influence on treatment outcomes, prognosis, and survival.^20^, ^21^ It is widely accepted that malnutrition takes a very important place in immune system, but malnutrition influences the immune functions which are fundamental to prevent infection or cancer through the cell‐mediated mechanism or other immune pathways.^22^, ^23^ Many studies have showed that preoperative malnutrition has a negative effect on the survival outcomes in patients with urologic carcinomas,^20^, ^24^ but few studies are performed to investigate the influence in UTUCs.^25^, ^26^

The PNI could be calculated by serum albumin level and lymphocyte count, both of which were routinely assessed and can be easily obtained by urologists before surgery. It is well accepted that lymphocyte plays an important role in cell‐mediated immunity in several cancers. As a result, the lymphocyte count could be a predictor of the survival. Serum albumin is also a simple marker for estimating the protein levels, which is usually used as a predictor of nutritional status. Gupta and colleagues^27^ investigated the connection between the serum albumin level and the treatment outcomes of patients with various cancers. Therefore, serum albumin levels are useful prognostic factors in malignant tumors.

In our study, the cutoff of PNI was calculated by the ROC curve analysis, and the mean value of PNI was lower than that in patients with renal cell carcinoma (RCC)^18^ but higher than that in patients with esophageal carcinoma.^11^ This finding shows that malnutrition is more common in gastrointestinal malignancy compared with UTUCs, and bad appetite and gastric obstruction may be the main reasons. As for the malnutrition in RCC is less common than that in UTUC, the age with the peak incidence in patients with UTUC is older than those with RCC might account for this.

In addition to PNI, the tumor stage, grade, size, architecture, variant histology, and lymph node invasion are proved as independent predictors in UTUC. Many of them have been recommended as prognostic factors by European Association of Urology guidelines and used for risk stratification except PNI.^1^ It may be because there are scarce studies concerning the prognostic value of PNI in UTUC. Even though the pathologic indicators, such as tumor stage, sessile carcinoma, positive lymph node, and CVH have higher HR than PNI, they just could be obtained via invasive therapy or after surgery. Conversely, we can calculate the PNI easily and rapidly from the preoperative laboratory examination results. Meanwhile, the blood test is cheaper than image examination, which could be used to estimate the tumor size. In addition, if the PNI could be recommended as a useful clinical reference, preoperative therapy such as neoadjuvant chemotherapy could be adopted to improve the outcomes. Therefore, we conducted this study to identify the independent predictors in UTUC, trying to provide more evidence for the risk stratification in UTUC.

A few limitations of our study should be noticed. First, it was a retrospective single center study, so the selection and information bias might not be avoided. Besides, some specific inflammatory indicator like cytokines and CRP were not routinely tested for the patients with UTUC, so we could not estimate their prognostic value. Furthermore, more high‐quality studies with long follow‐up time are still needed to provide more evidences for the prognostic value of PNI in patients with UTUC.

## 5 CONCLUSION

In conclusion, patients with low PNI had worse OS, CSS, and RFS. PNI is an independent predictor of oncologic outcomes in patients with localized UTUC after RNU. Therefore, we recommended that PNI could be incorporated in the traditional prognostic model, as an important predictor for the patients with UTUC.

## CONFLICT OF INTEREST

None declared.

## ETHICAL APPROVAL

All procedures conducted in our study involving human participants were consistent with the ethical standards of institutional and/or national research committee and with the Helsinki Declaration in 1964 and its subsequent amendments or similar ethical standards. For this type of research, there is no need for formal consent.
